# Prominent Role of Histone Modifications in the Regulation of Tumor Metastasis

**DOI:** 10.3390/ijms22052778

**Published:** 2021-03-09

**Authors:** Mariam Markouli, Dimitrios Strepkos, Efthimia K. Basdra, Athanasios G. Papavassiliou, Christina Piperi

**Affiliations:** Department of Biological Chemistry, Medical School, National and Kapodistrian University of Athens, 11527 Athens, Greece; myriam.markouli@gmail.com (M.M.); smd1700150@uoa.gr (D.S.); ebasdra@med.uoa.gr (E.K.B.)

**Keywords:** metastasis, epigenetics, histone modifications, EMT, acetylation, methylation, anoikis resistance, intravasation, drug targeting, novel therapies

## Abstract

Tumor aggressiveness and progression is highly dependent on the process of metastasis, regulated by the coordinated interplay of genetic and epigenetic mechanisms. Metastasis involves several steps of epithelial to mesenchymal transition (EMT), anoikis resistance, intra- and extravasation, and new tissue colonization. EMT is considered as the most critical process allowing cancer cells to switch their epithelial characteristics and acquire mesenchymal properties. Emerging evidence demonstrates that epigenetics mechanisms, DNA methylation, histone modifications, and non-coding RNAs participate in the widespread changes of gene expression that characterize the metastatic phenotype. At the chromatin level, active and repressive histone post-translational modifications (PTM) in association with pleiotropic transcription factors regulate pivotal genes involved in the initiation of the EMT process as well as in intravasation and anoikis resistance, playing a central role in the progression of tumors. Herein, we discuss the main epigenetic mechanisms associated with the different steps of metastatic process, focusing in particular on the prominent role of histone modifications and the modifying enzymes that mediate transcriptional regulation of genes associated with tumor progression. We further discuss the development of novel treatment strategies targeting the reversibility of histone modifications and highlight their importance in the future of cancer therapy.

## 1. Introduction

Cancer is associated with increased incidence and mortality worldwide due to complex reasons reflecting population age and growth as well as the prevalence of main cancer risk factors. Cancer prognosis is challenging to generalize and depends on host and tumor heterogeneity, including cancer subtype, tumor staging and grading that often associate with infiltration, aggressiveness, and dismal prognosis. Tumor stage is generally more informative than grade in determining prognosis and is based on the assessment of tumor size with direct invasion into nearby structures, lymph node involvement, as well as metastasis which commonly correlates with worst patient outcome.

Metastasis involves the growth of secondary tumors at distant sites from the primary location and is tightly connected to a well-coordinated gene regulation machinery. For metastasis to occur, cancer cells need to undergo epithelial to mesenchymal transition (EMT) characterized by loss of cell adhesion proteins, such as vimentins and cadherins, adapting a nonpolarized, spindle-shaped, and fibroblast-like appearance [1]. This further leads to loss of cell-to-cell and cell-to-matrix interactions, allowing neoplastic cells to escape from the primary site into the circulation. Intravasation requires resistance to anoikis, a form of apoptosis induced by loss of cell-to-cell and cell-to-matrix interaction, thus promoting the survival of neoplastic cells inside the blood vessels [2]. Under physiological conditions, cells anchored to extracellular matrix or to adjacent cells are equipped with a pro-survival machinery that can overwhelm apoptotic signals and promote normal cell function. Loss of anchorage allows cancer cell to overcome apoptosis and travel to a distal location [3]. Extravasation and colonization of the new tissue represent the final steps of the metastatic process. The ‘homing’ of cancer cells at target tissues is a highly specific process depending on adhesion and extravasation as well as the properties of tissue microenvironment [4].

**Figure 1:**
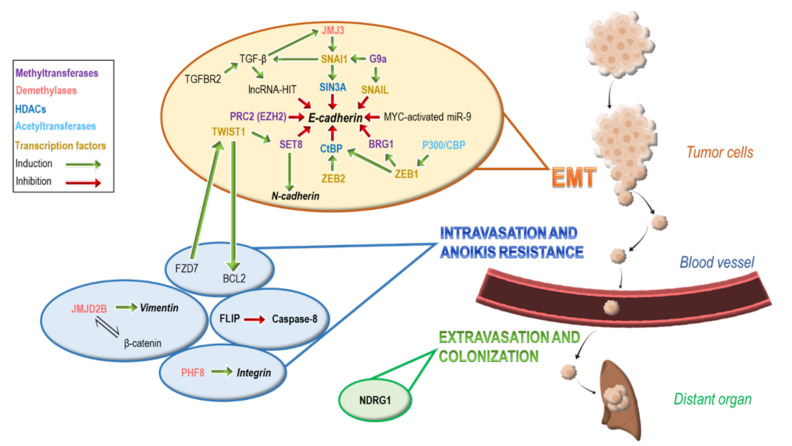
Epigenetic mechanisms regulate critical checkpoints of metastasis. Metastasis checkpoints are regulated by highly coordinated epigenetic mechanisms. EMT is mainly characterized by loss of E-cadherin expression. This can be achieved through regulation of transcription factors ZEB1, 2, SNAIL, and TWIST1. The p300/CBP complex acetylates the promoter of ZEB1, which further activates BRG1, in order to suppress E-cadherin via histone methylation. Additionally, ZEB1/ZEB2 have the ability tο recruit the CtBP complex, which enables HDAC1 to suppress the E-cadherin gene. At the same time, TGF-β, which is activated by TGFBR2-mediated induction of SNAI1/2, can activate both JMJ3 demethylase and lncRNA-HIT. JMJ3 is part of a loop along with TGF-β and SNAI1, regulating each other’s induction. Histone methyltransferase G9 targets SNAI1 which recruits SIN3A to suppress E-cadherin via deacetylation. Additionally, SET8 monomethylates H4K20 and cooperates with TWIST1 to increase N-cadherin and suppress E-cadherin. The methyltransferase EZH2, part of the PRC2 complex and the MYC-activated miR-9 can also inhibit E-cadherin expression by histone methylation and targeting of the E-cadherin mRNA. During the second step of intravasation and anoikis resistance, cancer cells express anti-apoptotic factors, such as BCL2 as well as mesenchymal markers. FZD7 induces activation of TWIST1 which increases BCL2 and FLIP inhibits anoikis by suppressing caspase-8 production. Moreover, JMJD2B interacts with β-catenin to upregulate the mesenchymal marker vimentin and PHF8 is involved in elevation of integrins expression. In the final step of extravasation and colonization, the cell differentiation *NDRG1* gene plays a crucial role in reversing EMT and promoting metastasis.

An interplay of genetic and epigenetic changes has been associated with the hallmarks of metastasis, representing critical regulators of genes required for cancer spread in a specific and reversible manner [5] (Figure 1). Epigenetic gene regulation can be achieved by chemically modifying DNA or histones, or by non-coding RNAs, such as microRNAs (miRNAs) and small interfering RNAs (siRNAs) [6]. Proper DNA and chromatin folding relies on histones integrity, stabilisation, and condensation. Increased condensation is associated with suppression of gene expression, whereas reduced condensation enhances gene activation. This process is highly dependent on epigenetic post-translational modifications (PTMs) taking place on the N- and C-terminals of histone proteins or on DNA. Histone modifications are broadly categorized into active or repressive, based on their effects on gene expression. Activating histone modifications include mainly acetylation, demethylation, and, in some cases, methylation of certain histones [7]. On the other hand, repressive modifications include histone methylation, deacetylation, and, less often, demethylation [7]. On top of these modifications, DNA methylation taking place on cytosines, mainly in CpG dinucleotides, induces further repression of gene expression. In parallel, non-coding RNAs act to alter gene transcription and further affect the post-transcriptional fate of the gene product [8]. In the following sections, we discuss main epigenetic mechanisms utilized by cancer cells to regulate gene expression, alter their transcriptional phenotype, and promote metastasis, focusing particularly on histone PTMs and their targeting options.

## 2. Epigenetic Modifications Regulate Epithelial to Mesenchymal Transition (EMT)

The EMT process can be activated by extracellular signals, such as extracellular matrix components, soluble growth factors such as the Transforming growth factor beta (TGF-β) and fibroblast growth factor (FGF), the signaling Wnt and Notch proteins, or by several intracellular cues which enhance signaling cascades, leading eventually to alterations in cytoskeletal organization and disassembly of cell–cell junction complexes [1,2,3]. A hallmark of EMT is the functional loss of the adhesion molecule E-cadherin present in most epithelial cells that leads to disaggregation of adjacent cancer cells and contributes to dissemination (Figure 1). The promoter of E-cadherin gene (*CDH1*) harbors E-box elements which are bound to transcription factors such as the Snail family of zinc finger proteins (SNAI1/2/3), the two double zinc finger and homeodomain factors ZEB1/2, and the family of bHLH factors (TWIST1/2, E12/E47)) that are ultimately involved in gene regulation [2,9]. Upon activation, these transcription factors act as molecular switches, induce transcriptional reprogramming, and mediate epithelial-mesenchymal plasticity, providing a rapid regulatory mechanism for cancer progression. Additional transcription factors such as the Forkhead Box C2 (FOXC2) and Transcription Factor 4 (TCF4) are involved in EMT without binding to *CDH1* but by suppressing the expression of claudins and desmosomes present in cell junctions, or through interaction with other transcription factors.

During transcriptional reprogramming, alterations in histone modifications play a pivotal regulatory role, orchestrating the simultaneous repression of epithelial genes and activation of mesenchymal genes. Based on their effects on gene transcription and the associated chromatin environment, they are broadly characterized either as permissive or repressive. Permissive PTMs may induce the upregulation of mesenchymal genes, such as *N*-cadherin (*CDH2*), VE-cadherin (*CDH5*), fibronectin (*FN1*), and vimentin, whereas repressive histone modifications mainly participate in the suppression of epithelial genes, such as *CDH1*, claudin 1 (*CLDN1*), claudin 10 (*CLDN10*)*,* and junction plakoglobin epithelial gene (*JUP*) [10].

### 2.1. Activating Histone Modifications Involved in EMT

**Figure 2:**
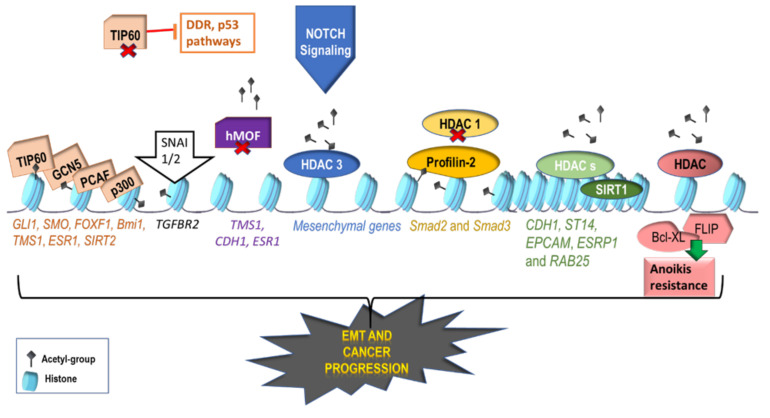
Histone acetylation regulates genes involved in cancer progression. Histone acetylation is an activating histone modification that favors gene transcription. H3K4Ac marks are present on the promoters of several genes, *GLI1*, *SMO*, *FOXF1*, *Bmi1*, and *SIRT2*, which are upregulated in cancer, favoring tumor progression. Upon SNAI1/2 activation, increased H3K9 acetylation has been observed on the promoter of *TGFBR2*, causing its upregulation. Critical acetyltransferases TIP60, GCN5, PCAF, and p300 catalyze histone acetylation of metastasis-promoting genes. hMOF, a H4K16 histone acetyltransferase normally maintains the expression of EMT-related tumor suppressor genes, such as *TMS1, CDH1*, and *ESR1*, but is often downregulated in cancer. TIP60 is also commonly downregulated in various tumors, where it prevents the activity of anti-tumor DDR and p53 pathways, indicating how reduction in histone acetylation promotes cancer progression. HDAC3 can also be detected on the promoters of activated mesenchymal genes, even though most HDACs are associated with gene repression. Upon Notch signaling activation, H3K4Ac is removed by HDAC3, allowing for Notch-mediated expression of EMT-related genes. Profilin-2 interacts with HDAC1 and inhibits its binding to the promoters of *Smad2* and *Smad3*, causing Smad protein activation and subsequently enhancing TGF-β-induced EMT and angiogenesis. HDACs also catalyze H3K4/56 deacetylation at the *CDH1* promoter, thus repressing E-cadherin. Similarly, HDAC1 and 2 are recruited by ZEB1 to *CDH1* promoter, inducing its repression. In the same context, ZEB1 recruits SIRT1, a nicotinamide adenine dinucleotide (NAD)-dependent deacetylase, associated with a global H3K27 deacetylation and reduced H3K9Ac and H3K27Ac levels on the promoters of *CDH1* and other epithelial genes, such as *EPCAM*, *ST14, ESRP1*, and *RAB25*, promoting EMT and metastasis. Finally, overexpression of FLIP leads to the upregulation of the anti-apoptotic Bcl-XL, inhibiting the anoikis apoptotic pathway. HDAC inhibition prevents this outcome by reducing FLIP levels and inducing apoptosis of cancer cells, suggesting that HDACs participate in anoikis resistance of cancer cells through yet unknown mechanisms.

Activating histone PTMs include mainly acetylation and demethylation. It is well-established that actively transcribed euchromatin is mainly characterized by high lysine acetylation levels catalysed by the acetylases Lysine Acetyltransferase 2B (PCAF), Histone Acetyltransferase P300/CREB Binding Protein (p300/CBP), and Tat Interacting Protein, 60 kDa (TIP60) (Figure 2).

During EMT, β-catenin, which is a core component of the cadherin protein complex, has been shown to interact with the T cell factor, in order to translocate to the nucleus and recruit the p300/CBP complex [11]. This further induces acetylation of the transcription factor ZEB1, enhancing its binding to the microRNA-200c/141 promoter, ultimately leading to its expression [12].

Histone 3 lysine 4 acetylation (H3K4Ac) marks mediated by TIP60, are often detected in the promoters of EMT marker genes, including *CDH1*, glioma-associated oncogene homolog 1 (*GLI1*) and smoothened homolog precursor (*SMO*) of the Hedgehog pathway which are involved in cancer cell migration and invasion [13]. H3K4Ac marks are also present in the transcription regulators Forkhead Box F1 (FOXF1) and BMI1 Proto-Oncogene, Polycomb Ring Finger (BMI1), that promote tumor progression and stemness [13,14].

Increased H3K9 acetylation was observed on the promoter of Transforming Growth Factor Beta Receptor 2 (*TGFBR2*) after the activation of SNAI1/2, leading to transcription of TGF-β, a major player in the regulation of cancer metastasis [15]. In addition, acetylation of H4K16, mediated by histone acetyltransferase Males-absent-on-the-first (hMOF/KAT8/MYST1) has been demonstrated to maintain the expression of EMT-related tumor suppressor genes, such as target of methylation-induced silencing (*TMS1*), *CDH1*, and Estrogen Receptor 1 (*ESR1*) [16]. hMOF is therefore often downregulated in breast cancer and medulloblastoma, resulting in genomic instability and impaired DNA damage response [16].

Another activating histone modification, demethylation is mainly mediated by the enzymes Jumonji Domain-Containing Protein 1A/B (JMJD1A/B), Jumonji Domain Containing 2 (JMJD2) and the PHD Finger Protein 2/8 (PHF2/8) [17]. JMJD1A has been shown to upregulate Metastasis Associated Lung Adenocarcinoma Transcript 1 (MALAT1), a long non-coding RNA that is involved in dysregulation of cell signaling and EMT induction, cancer cell migration and invasion [18]. Jumonji Domain Containing 2 B (JMJD2B) can interact with β-catenin to mediate H3K9 demethylation of the vimentin (VIM) gene promoter, and further promote EMT and metastasis [19]. Similarly, PHF8 causes H3K9 demethylation on the promoter of integrin genes including Integrin Subunit B2 (ITGB2), Integrin Subunit Alpha M (ITGAM), and Integrin Subunit Alpha 9 (ITGA9) to induce their expression [20]. Lastly, Jumonji Domain-Containing Protein 3 (JMJ3), a histone demethylase that removes methyl marks from H3K27, is induced by TGF-β and activates SNAI1 expression to facilitate EMT [21].

### 2.2. Repressive Histone Modifications Involved in EMT

**Figure 3:**
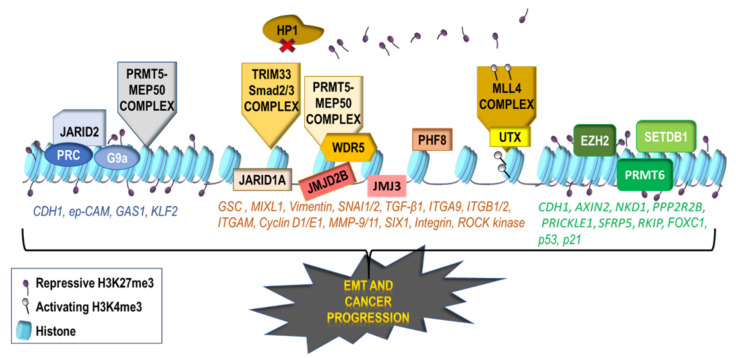
Role of histone methylation in gene regulation during tumor progression. Trimethylation of H3K27 and H3K4 has been associated with suppression of epithelial genes or activation of mesenchymal ones, respectively. In this process, the PRMT5-MEP50 complex suppresses *CDH1* and *GAS1* by mediating H3K4 and H4R3 methylation. E-cadherin is suppressed by SNAI1-mediated PRC2 trimethylation of H3K27. PRC2 also suppresses *KLF2* expression. JARID2 recruits both PRC2 and G9a to promote H3K27 and H3K9 methylation at the promoters of *CDH1* and *miR-200*. G9a also silences the *ep-CAM* gene, promoting metastasis. The TRIM33/Smad2/3 complex inhibits the binding of HP1 on the DNA, leading to expression of *GSC* and *MIXL1* mesenchymal genes. The PRMT5-MEP50 complex recruits WDR5 and causes H3R2 methylation, activating the EMT-promoting genes *VIM* and *SNAI1/2*. Over-expression of the histone demethylase, JARID1A leads to the activation of cyclin D1/E1 and ITGB1 expression, promoting tumor progression. The H3K27 demethylase, UTX, activates several pro-metastatic genes, such as *MMP-9/11* and *SIX1*, after interacting with the MLL4 complex, which includes a H3K4 methyltransferase, thus causing enhancement of EMT and metastasis. The PHF8 demethylase upregulates the expression of Vimentin, Integrin, and Rho-associated protein kinase (ROCK) kinase by removing H3K9 methylation marks, thus favoring metastasis. JMJD2B, a H3K9 demethylase, induces the expression of Vimentin, after interacting with β-catenin and also demethylates the Integrin *(ITGB2, ITGAM, ITGA9, ITGAB2)* gene promoters, further promoting EMT and metastasis. JMJ3, a H3K27 histone demethylase is induced by TGF-β and activates SNAI1 expression to facilitate EMT. EZH2 establishes repressive H3K27me3 marks and downregulates *FOXC,* inducing metastasis. It also silences the *CDH1,* *AXIN2, NKD1*, *PPP2R2B*, *PRICKLE1*, *SFRP5*, *RKIP*, promoting EMT and cancer metastasis. SETDB1 methyltransferase amplification is responsible for cancer cell growth by methylating and stabilizing the oncogenic p53 mutants. In this context, PRMT6 methyltransferase reduces the expression of p21, promoting tumor cell migration.

Repressive histone modifications mainly involve methylation and deacetylation. Histone methylation occurs on arginine and lysine residues by histone methyltransferases (HMTs) which use S-adenosyl methionine (SAM) as a donor for methyl groups [8] (Figure 3). The Enhancer of zeste homolog 2 (EZH2) methyltransferase, part of the repressive Polycomb Repressive Complex 2 (PRC2) complex has been overexpressed in cancer and especially in metastatic tumors [22]. EZH2 mediates the H3K27me3 marker which has been associated to silencing of the *CDH1* gene, *Wnt* antagonist genes such as Axis Inhibition Protein 2 (*AXIN2*), Naked1 (*NKD1*), Protein Phosphatase 2 Regulatory Subunit 2 beta (*PPP2R2B*), Prickle Planar Cell Polarity Protein 1 (*PRICKLE1*), Secreted Frizzled Related Protein 5 (*SFRP5*) and of the metastasis suppressor gene Raf Kinase Inhibitory Protein (*RKIP*) [22], promoting EMT and cancer metastasis [23] (Figure 3). The expression of PRC2 complex on the other hand has been inversely linked to metastasis and PRC2 loss has been involved in the enhancement of tumor metastatic potential [24].

The methyltransferase G9a that mediates H3K9me2 can interact with SNAI1 and promote EMT by silencing a wide array of genes [25]. Furthermore, it can recruit DNA methyltransferases (DNMT) on the promoter of *CDH1* through SNAIL interaction, further suppressing E-cadherin expression and promoting EMT in vitro and in vivo [26]. Hypoxia promotes G9a stability and promotes indirectly tumor progression [27]. The effects of G9a can be partly attributed to its interaction with SNAI1, which induces EMT by silencing a wide array of genes [25] (Figure 3). 

Another histone methyltransferase SET Domain Bifurcated Histone Lysine Methyltransferase 1 (SETDB1) mediates the repressive H3K9me3 mark and inhibits gene transcription. It has been shown to potentiate EMT by up-regulation of Signal Transducer And Activator Of Transcription 3 (STAT3) and induction of TWIST and c-myc [28]. Additionally, SETDB1 cooperates with ΔNp63α to induce its stabilization, and ΔNp63α recruits SETDB1 to target genes that are going to be repressed [29]. However, SETDB1 can also interact with Mothers Against Decapentaplegic Homolog 2/3 (SMAD2/3) to suppress EMT and metastasis [30].

Moreover, the histone methyltransferase SET Domain Containing Lysine Methyltransferase 8 (SET8) can monomethylate H4K20 and cooperate with TWIST to reduce *CDH1* and upregulate *CDH2* in a mouse model in vitro [31]. Di- and tri-methylation of H4K20 is mediated by the methyltransferase Suppressor Of Variegation 4-20 Homolog 1/2 (SUV420H1/2), commonly lost in many tumors where it affects chromatin integrity [32,33] and regulates tensin 3 (TNS3) adhesion protein [34].

A subgroup of ATP-dependent chromatin remodeling complexes, the SWItch/Sucrose Non-Fermentable (SWI/SNF) comprised of two catalytic subunits, BRM/SWI2-Related Gene 1 (BRG1) and BRG1-Associated Factor 60C (BAF60c) has been involved in EMT and tumor progression. BRG1 is recruited by ZEB1 on the *CDH1* promoter inducing gene silencing [35], while BAF60c upregulates WNT5a, a member of WNT signaling, activated during EMT [36].

In addition, the Snail family of transcription factors can recruit the lysine-specific demethylase 1 (LSD1) which catalyzes H3K4me2 demethylation, acting downstream to silence Snail-regulated genes, such as *CDH1*. Transcription factors that activate Snail also interact with Histone Deacetylase 1 (HDAC1), HDAC2 [37], and PRC2 [1]. Snail has been also implicated in the formation of bivalent chromatin state harboring both repressive H3K27me3 marks as well as active H3K4me3 marks. Bivalent genes exist in a poised state, which makes them readily available for activation upon stimulation [38]. The property of bivalency is of major importance in cancer metastasis, contributing to EMT reversibility.

The other repressive histone PTM, deacetylation involves the removal of permissive marks from histones, inhibiting gene expression. Histone deacetylases (HDACs) can form large complexes that are recruited to specific areas, decreasing gene expression. SNAI1, for instance, can recruit the SIN3 Transcription Regulator Family Member A (SIN3A) repressor complex on the *CDH1* promoter [39]. This complex contains HDAC 1/2 capable to remove the acetyl marks in gene promoter and decrease its expression. Moreover, SNAI2 can form a complex with the evolutionarily conserved SNAG (Snail/Gfi) domain and bind on the E-box of the *BRCA2* promoter, where it recruits the oncogenic transcriptional corepressor C-terminal-binding protein (CtBP) complex that contains HDAC1, further inducing gene repression [40]. ZEB1/2 has been shown to recruit this complex on the promoter of *CDH1* [41]. Furthermore, HDAC6 and Sirtuin 1 (SIRT1) have been shown to counteract the p300-mediated acetylation on cortactin and thus enhance its ability to bind F-actin, leading to induction of EMT and tumor progression [42].

### 2.3. DNA Methylation Involved in EMT

Aberrant DNA methylation is often observed in cancer, mediated by DNA methyltransferases (DNMTs) which are responsible for the formation of methylome, a dynamic chromatin state where genes can be silenced or activated in a quick, specific way, without altering their DNA sequence [43]. Upon induction of EMT, the methylome undergoes specific changes which alter the expression profile of cancer cells and promote the transcription of EMT-associated genes [44]. Silencing of *CDH1* during EMT has been associated with promoter hypermethylation, induced by the transcription factors SNA1 [26] and ZEB1 [45] that recruit DNMTs. The DNMT1 cross-talks with the TGF-β/SMAD2 pathway [46] and along with DNMT3A play a significant role in silencing the miR-200 family members (miR-200b, miR-200a, miR-429, miR-200c, and miR-141) during EMT [47,48,49]. DNMTs also recruit methyl-DNA-binding domain proteins such as Methyl-CpG Binding Protein 2 (MeCP2) and Methyl-CpG Binding Domain Protein 1-4 (MBD1-4) [50] that mediate chromatin compaction upon DNA methylation [51]. Methylation is also a reversible event with DNA demethylases such as the three Ten-Eleven Translocation (TET) proteins, creating the dynamic setting mentioned above [52].

### 2.4. Non-Coding RNAs (Nc-RNAs) Involved in EMT

Non-coding RNAs (ncRNAs) are also involved in the regulation of gene transcription and EMT. MicroRNAs (miRs), a subtype of ncRNAs, target mRNAs post-transcriptionally and promote degradation, inhibition of translation, or both [53]. During EMT, miR-200s, miR-205, and ZEB1/2 act to suppress each other, forming an interactive loop [54]. In this loop, miRs were shown to suppress EMT while ZEB1/2 promotes EMT and cancer progression [55]. TGF-β2 and β-catenin are also repressed by miR-200s, thus inhibiting the EMT process [56]. Moreover, miRs have been shown to target other pro-EMT genes, including *EZH2*, *SNAI1/2*, *TWIST1/2* [57,58]. Additional miRNAs, such as the *Myc*-activated miR-9, can promote EMT through targeting of the *CDH1* gene [59] (Figure 1).

Long non-coding RNAs (lncRNAs) have also been shown to contribute to EMT and tumor progression through several pathways [60]. LncRNA homeobox (HOX) Transcript Antisense RNA (HOTAIR) that has been associated to metastasis and poor prognosis in several cancer types [61], can interact with both PRC2 and LSD1 in order to repress *HOX* genes such as *HOXD8*, *HOXD9*, *HOXD10*, *HOXD11* along with other metastasis suppressor genes, such as P53-Responsive Gene 1 (*PRG1*), Junctional Adhesion Molecule 2 (*JAM2*), Protocadherin 10 (*PCDH10*), and Protocadherin Beta 5 (*PCDHB5*) [62]. Furthermore, TGF-β signaling may induce lncRNA-HIT (HOXA transcript induced by transforming growth factor (TGF)-β), which in turn inhibits the expression of E-cadherin, thus promoting EMT and the metastatic process [63]. Collectively, these data depict the complexity of epigenetic regulation that uses several different repressive methods to achieve quick control of gene expression levels.

## 3. Epigenetic Modifications Involved in Intravasation and Anoikis Resistance

Intravasation and anoikis resistance are the next crucial steps in the process of metastasis. Cancer cells that have undergone EMT and have lost their anchorage, need to suppress apoptotic signals. These signals act by promoting the death receptor activated pathway which involves activation of the initiator caspase-8 along with several other caspases [64]. Cancer cells are able to block this pathway by overexpressing the FLICE-like inhibitory protein (FLIP) which inhibits caspase-8, and is associated with worse prognosis [65] (Figure 1). Moreover, anoikis resistance is also regulated by the Frizzled Class Receptor 7 (FZD7)-TWIST1 interaction where FZD7 regulates the expression of TWIST1 via histone PTMs, such as H3K4me3 and H3K27Ac. These activating modifications increase the expression of transcription factor TWIST1, as validated by the reduction of TWIST1 expression after FZD7 knockdown [66]. BCL2 levels were shown to increase in parallel to TWIST1 levels. Both FZD7 and BCL2 are correlated with the Wnt pathway, and the knockdown of WNT5A, a ligand of FZD7, was shown to decrease BCL2 expression [66].

## 4. Epigenetic Modifications Involved in Extravasation and Colonization

The last steps in metastasis, extravasation, and colonization require circulating cancer cells to alter again their phenotype in order to ‘home’ new tissues. In this process, cancer cells are required to attain an epithelial-like phenotype which is achieved by reversing EMT in a process called mesenchymal to epithelial transition (MET). This allows the cancer cells to express adhesion molecules and cytokines which crosstalk with the cells of the local tissue and induce recruitment of immune cells [67]. In this way, cancer cells orchestrate their extravasation [68] by upregulating the expression of integrins and selectins [69], leading to elevated cadherin expression [70]. Epigenetic mechanisms are also implicated in this final step of metastasis mainly through histone methylation. Regulation of the cell differentiation *N*-Myc-Downstream Regulated gene 1 (*NDRG1*) that induces MET change and is involved in cancer cell metastasis, can be achieved through histone methylation marks [71] (Figure 1). Notably, in cell lines with increased expression of NDRG1 and low metastatic potential, increased methylation of H3K4 has been detected in *NDRG1* gene. Importantly, *NDRG1* is often silenced in most metastatic cancers [72].

## 5. Cancer-Type Specific Histone Modifications Regulating Tumor Metastasis

Specific epigenetic changes have been associated with the regulation of metastasis and the acquisition of invasive phenotype in several tumors, as described in this section.

### 5.1. Head and Neck Cancer

Acetylation of H3K4 has been detected on the promoters of EMT marker genes, including *CDH1*, *GLI1*, and *SMO* which are involved in cell migration and invasion in head and neck squamous cell carcinoma, serving as prognostic markers [13]. Of interest, HDAC3 has also been detected on the promoters of activated mesenchymal genes. It has been shown to remove H3K4Ac from specific gene repressing transcription factors such as Ikaros, allowing the upregulation of EMT genes through activation of Notch signaling, and enhancing metastasis [13].

### 5.2. Lung Cancer

In lung cancer, H3K4Ac marks have been detected in the transcription regulators FOXF1, and Bmi1 that promote tumor progression and stemness [13,14]. FOXF1 induces EMT through upregulation of lysyl oxidase (LOX) that is critical for the crosslinking of extracellular matrix proteins as well as through suppression of Smad2/3 signaling or transcriptional activation of SNAI1. Moreover, the actin binding protein profilin-2 interacts with HDAC1 to inhibit its binding to the promoters of *Smad2* and *Smad3*, leading to Smad protein activation and subsequently enhancing the TGF-β-induced EMT and angiogenesis in lung cancer cells [73].

Apart of acetylation, histone methylation has also been observed in lung cancer cells. In non-invasive tumor cells, TGF-β favors the association of methyltransferase SETDB1 with Smad3 which mediates H3K9me in the *Snail* promoter, repressing its expression [74]. On the other hand, in invasive tumor cells undergoing TGF-β-induced EMT, repression of SETDB1 causes de-repression of the *Snail* promoter [74]. The association of SETDB1 with Smad3 has been also shown to suppress metastasis in lung cancer by repressing *IL-2* and the Ca^2+^-dependent RNA-binding protein annexin A2 (*ANXA2*) that interacts with the mRNA of the nuclear oncogene, *c-myc* [30,75].

Additionally, H3K9me3 in the Tripartite Motif Containing 33 (TRIM33)-Smad2/3 complex displaces the heterochromatin protein 1 (HP1), allowing the subsequent activation of mesendoderm differentiation genes, such as Goosecoid Homeobox (*GSC*) and Mix Paired-Like Homeobox (*MIXL1*) [76,77].

TGF-β can also activate the Jumonji and AT-Rich Interaction Domain Containing 2 (JARID2), a component of the PRC2 complex that downregulates E-cadherin expression in lung cancer cells. JARID2 occupies the promoters of *CDH1* and *miR-200* family members and controls the recruitment of PRC and G9a methyltransferase, promoting methylation of H3K27, H3K9, and gene repression [78]. The pro-metastatic effect of G9a observed in lung cancer is attributed to gene silencing of epithelial cell adhesion molecule *(ep-CAM*), enhancing invasion [79].

Moreover, the protein arginine methyltransferase 5 (PRMT5) forms complex with the methylosome protein 50 (MEP50) to catalyze histone mono- and dimethylation of important EMT genes in lung cancer cells [80]. Specifically, the PRMT5-MEP50 complex mediates H3R2me through recruitment of the nuclear scaffolding protein WD Repeat Domain 5 (WDR5), causing the activation of EMT-promoting genes, such as *VIM*, Snail and Slug *(SNAI1/2)*. They also mediate H3K4 and H4R3 methylation to repress metastasis suppressing genes, such as *CDH1* and the Growth arrest-specific 1 (*GAS1*) [80]. Additionally, overexpression of a H3K4 histone demethylase, Jumonji, AT Rich Interactive Domain 1A (JARID1A) leads to the activation of *cyclin D1/E1* and Integrin Subunit Beta 1 *(ITGB1)* expression, promoting lung cancer cell growth and metastasis [81].

In parallel with histone modifications, DNA methylation is also implicated in lung cancer metastasis. Overexpression of the programmed death-1 (PD-1) ligand 1 (PD-L1) that regulates tumor microenvironment during EMT has been attributed to decreased DNMT1 levels in lung cancer cells which lead to demethylation of the *PD-L1* promoter, changing its expression [82].

### 5.3. Breast Cancer

Acetylation marks (H3K4Ac) are present in estrogen receptor (ER) signaling-responsive genes in breast cancer cells and their detection has proved useful in predicting early stages of tumor progression [83]. The acetyltransferase TIP60 that establishes H3K4, is involved in oncogene-induced DNA damage response and is often downregulated during hypoxia-induced EMT in ER-negative tumors. Reduced TIP60 expression was shown to block anti-tumor responses, including the DNA Damage Response (DDR) and p53 pathway, suggesting that H3K4Ac reduction favors ER-negative breast cancer progression [84]. On the other hand, TIP60 depletion in ER-positive breast cancer inhibits tumor development. This is possibly attributed to TIP60 recruitment by estrogens, promoting gene transcription. TIP60 interaction with ER-α results in the recruitment of histone methyltransferase Mixed Lineage Leukemia 1 (MLL1), which increases the H3K4me and H2AK5Ac marks, activating target genes that participate in the development of breast cancer [84].

The histone acetyltransferase General Control Of Amino Acid Synthesis Protein 5-Like 2 (GCN5) plays a critical role in the TGF-β/Smad signaling pathway in breast cancer cells [85]. GCN5 inhibition prevents EMT, migration and invasion of breast cancer cells. Another acetyltransferase family member, PCAF promotes EMT and cancer metastasis [86]. The p300 acetylase has been shown to interact with a complex comprised of the Disruptor of telomeric silencing 1-like (DOT1L) histone lysine methyltransferase and *c-Myc*, activating EMT regulators in breast cancer, and serving as a potential oncogene responsible for the formation of an aggressive phenotype and transformation of cancer stem cells (CSCs) [87].

Histone deacetylases are also highly involved in breast cancer metastasis. HDAC1 induces the growth and migration of breast cancer cells by upregulating Snail/IL-8 signals [88]. HDAC2 enables the motility of breast cancer cells by upregulating Matrix Metalloproteinase 2 (MMP2) and *N*-cadherin and HDAC3 associates with Epidermal Growth Factor Receptor (EGFR) and c-Src to promote breast cancer cell invasion [89,90]. In contrast, HDAC8 forms a complex with SMAD3/4, in order to transcriptionally suppress SIRT7 and thus inhibit metastasis, increasing the efficacy of chemotherapy in breast cancer [91]. Finally, SIRT7 reduces breast cancer metastasis by degrading SMAD4, the key factor in TGF-β pathway, thus inhibiting this pathway [91].

Moving towards methylation, the histone methyltransferase PRMT1 was shown to bind *ZEB1* promoter and induce EMT in breast cancer cells [92]. Similarly, PRMT7 regulates E-cadherin expression and promotes EMT [93] whereas PRMT6 attenuates p21 signaling [94]. Additionally, PRMT9 activates the Phosphoinositide 3-kinase/AKT Serine/Threonine Kinase/Glycogen Synthase Kinase 3 Beta/Snail (PI3K/Akt/GSK-3b/Snail) pathway, enhancing cell migration and invasion [95].

In contrast, the methyltransferase SUV420H2 attenuates EMT and tumor progression by establishing H4K20me3, and decreasing the expression of the focal adhesion protein tensin-3 (TNS3) [34].

The lysine-specific demethylase JARID1A, upregulates the expression of the extracellular matrix protein Tenascin C (TN-C), and favors breast cancer cell invasion [96]. The demethylase JARID1B, promotes EMT by suppressing miR-200s [97] or Phosphatase And Tensin Homolog (PTEN) [98] through H3K4 demethylation of its promoter. However, JARID1B can act in concert with LSD1, to remove three H3K4 methylation marks from the C-C Motif Chemokine Ligand 14 (*CCL14*) promoter, resulting in inhibition of chemokine-mediated migration, angiogenesis and breast cancer metastasis [99].

The Ubiquitously transcribed tetratricopeptide repeat, X chromosome (UTX/KDM6A) demethylase activates several oncogenes and pro-metastatic genes, such as metalloproteinases *MMP-9/11* and Homeobox protein Sineoculis homeobox homolog 1, Sine Oculis Homeobox Homolog 1 (*SIX1*), upon interaction with the H3K4 methyltransferase, MLL4 complex, enhancing EMT and breast cancer metastasis [100]. However, UTX has been also shown to cooperate with LSD1, HDAC1 and DNMT1 to compete with the MLL histone methyltransferase complex and disrupt the recruitment of c-Myc and p300. This induces the inhibition of H3K4 methylation on the promoters of EMT regulators, including *Snail*, *ZEB1*, and *ZEB2* [101], as well as on the promoter of *CDH1*.

At this point, it is important to note that different histone PTMs are associated with specific breast cancer subtypes. H3K9ac and H3K36me3 marks are often detected in HER2-positive breast cancer cells, while H3K9Ac, H3K4me3 and H3K79me2 are predominant in triple negative breast cancer (TNBC) [102]. H3K27me3 is present in lower grade tumors, Luminal A and B1 subtypes [103,104] where it associates with repression of the Forkhead Box C1 (*FOXC1*) gene in Luminal B breast cancer, resulting in metastatic behavior [105]. SIRT1 is upregulated in luminal and HER2-positive subtypes but is significantly downregulated in TNBC, whereas H3K4ac, H3K9ac, and H4K16ac are relatively upregulated in TNBC but greatly reduced in luminal and HER2-positive subtypes [106].

### 5.4. Gastrointestinal Cancers

Several deacetylases have a predominant role in gastrointestinal cancers. SIRT2 is involved in the regulation of Akt/GSK-3β/β-catenin pathway to mediate EMT and associates with prognosis in hepatocellular carcinoma and esophageal squamous cell carcinoma. In hepatoma cells, HDACs catalyze H3K4/56 deacetylation at the *CDH1* promoter, inducing E-cadherin repression by Snail2, in favor of EMT [107]. HDAC1/2 are recruited to ZEB1 on *CDH1* promoter, inducing its repression in pancreatic cancer cells [41]. Moreover, overexpression of FLIP has been shown to mediate a concomitant overexpression of the anti-apoptotic factor Bcl-XL, and inhibit the anoikis apoptotic pathway in a pancreatic cancer cell model [108]. This result was counteracted by HDAC inhibition, which reduced FLIP levels and induced apoptosis of cancer cells, suggesting that HDACs participate in anoikis resistance of cancer cells [108].

Furthermore, amplification of the SETDB1 methyltransferase is responsible for the outgrowth of liver cancer cells by methylating and stabilizing the oncogenic p53 mutants [109].

The PRC2 complex that establishes repressive H3K27me3 marks has been shown recruited on the Kruppel Like Factor 2 (*KLF2*) and *CDH1* promoters in gastric cancer cells [110]. Α switch of active H3K4me3 to repressive H3K27me3 has been detected on 102 EMT marker gene promoters, whereas the opposite switch of H3K27me3 to the activating H3K4me3 was observed on a series of upregulated mesenchymal gene promoters, such as *ZEB2, CDH2,* Platelet Derived Growth Factor Receptor α *(PDGFRα)* and *ESRP1* in a TWIST1-induced EMT cell model [111]. Additionally, a third set of promoters containing both H3K4me3 and H3K27me3 marks were detected to regulate several bivalent genes in the same model, indicating an increased level of plasticity of the mesenchymal cell state [111].

Finally, DNA methylation has been also shown to regulate *TWIST1/2* promoter methylation and correlate inversely with the TWIST1/2 expression levels in colorectal cancer. TWIST1/2-positive high-grade tumors exhibit both lymphatic vessel invasion and lymph node metastasis suggesting that *TWIST* promoter methylation may serve as a prognostic marker for patients with colorectal cancer [112]. Similarly, ZEB2 expression is also regulated by promoter methylation in pancreatic and hepatocellular carcinoma [113,114].

### 5.5. Prostate Cancer

Several histone modifications have been detected in prostate cancer cells with NAD-dependent deacetylase SIRT1 being recruited in ZEB1 to induce silencing of *CDH1* and promoting EMT [115]. ZEB1-induced EMT is accompanied by repression of other epithelial genes, such as Epithelial cell adhesion molecule (*EPCAM*), Suppressor of tumorigenicity 14 (*ST14*), Epithelial Splicing Regulatory Protein 1 (*ESRP1*), and *RAB25*, reduced H3K9Ac and H3K27Ac on their promoters and a global H3K27 deacetylation [116]. Additionally, TGF-β promotes H3K4me3 and Retinoblastoma-Binding Protein 5 (RbBP5) binding to the promoter of *Snail* by recruiting Smad2/3 and CBP, leading to enhanced Snail expression in prostate cancer cells [78]. Furthermore, PHF8 demethylase was demonstrated to remove H3K9 methylation on the promoter of integrin genes and Rho-Associated Protein Kinase 1 (*ROCK*) kinase in prostate cancer cells and inducing their expression and promoting migration and invasion, thus being correlated with poor prognosis [20].

## 6. Targeting Options

The high predominance of epigenetic changes in the metastatic process of several tumors, along with their reversibility, has pointed research efforts towards their therapeutic targeting for the development of new epigenetic drugs that will be patient- and cancer-subtype specific (Table 1).

### 6.1. Low Molecular Weight EMT Inhibitors

An interesting EMT therapeutic approach is the use of small molecular weight inhibitors of EMT promoting genes, which target specific binding proteins that recognize different histone modifications with promising results in vitro [117]. These include small molecule compounds that target the acetylated-histone binding bromodomain (BD), specifically blocking the binding to acetylated lysine by the BD-containing transcriptional co-activator, Bromodomain Containing 4 (BRD4) [118,119]. Since BRD4 is found in the super-enhancer region of the *Myc* oncogene, its inhibition prevents Myc function in tumor cells [120]. More specifically, treatment with the BRD4 inhibitor MS417 was shown to reduce metastasis of colorectal cancer in mouse models by altering the expression of key EMT genes, causing a sharp increase in the epithelial marker E-cadherin, as well as a decrease in the mesenchymal marker vimentin [121]. Another BRD4 inhibitor JQ1 along with MS417 were demonstrated to interfere with BRD4 binding to Keratin 73 (K73)/K76Ac2 on TWIST transcription factor and repress WNT5A expression, thus reversing EMT and metastasis of breast cancer in vitro and in vivo [122]. Currently BRD4 inhibitors are tested in multiple clinical trials of different cancer types. Other small molecules, such as UNC1215 and UNC3866 that target specific methylated histone binders have been also used, even though their function in reversing EMT and tumor progression has yet to be clarified. They were shown to block the binding of methyl-lysine by the Malignant Brain Tumor (MBT) domain-containing protein Lethal(3)Malignant Brain Tumor-Like Protein 3 (L3MBTL3) [123] and CD-containing protein Chromobox 4/7 (CBX4/7) [124], respectively. Furthermore, in the context of EMT, TGF-β1 treatment in gastric cancer cells induced expression of JARID1A demethylase, which is recruited by p-SMAD3 to *CDH1* promoter, inducing its silencing, and promoting malignant progression [125]. Finally, drugs, such as salinomycin that target CSCs, which play a great role in EMT have been successfully used in preclinical settings of various tumors [126,127,128,129,130]. Experimental data support that these epigenetic cancer treatments may influence the Twist-Snail/ZEB-E-cadherin axis and EMT inducers, such as Wnt-TGFβ-Bone Morphogenetic Protein (BMP). Finally, RNA interference techniques also seem to rise as a more promising approach for repressing these TFs on mRNA level [131].

### 6.2. Histone-Modifying Drugs

Several epigenetic drugs have already been approved for clinical use, including the combination regimen of DNA methyltransferase inhibitors (DNMTi) with histone deacetylase inhibitors (HDACi) in the treatment of myelodysplastic syndromes [132]. Clinical trials (up to phase IIb) with the same regimen have also been conducted for non-Hodgkin’s lymphomas, such as T-cell and diffuse large B-cell lymphoma, as well as acute myeloid leukemia [133]. In other solid tumors, for example non-small cell lung cancer, these drugs have yet to be established as a preferred treatment option and are only being used as experimental therapies in advanced, recurrent, or refractory states [133,134]. Moreover, panobinostat, a novel potent inhibitor of all HDAC enzymes has been effective towards cancer proliferation and apoptosis [135], as well as towards the expression of differentiation and EMT markers in in vivo hepatoma models [136]. Panobinostat was shown to upregulate the epithelial cytokeratin marker and downregulate the mesenchymal vimentin marker. The combination of the histone deacetylase inhibitor, Suberoylanilide hydroxamic acid (SAHA), and the methyltransferase inhibitor, Zebularine affects the differentiation of pancreatic cancer models [137]. Peroxiredoxin-2 (TSA) has also been used to target class I HDACs both in vivo and in vitro and resulted in reduced cancer cell growth along with EMT and metastasis suppression [138]. Mocetinonstat was also the only HDACi that demonstrated specific antagonism of ZEB1-mediated miR-203 repression in pancreatic cancer cells. miR-203 silencing by ZEB1 is strongly associated with tumor recurrence after treatment with gemcitabine [139]. A number of HDACi have also been investigated in the treatment of solid tumors, including breast cancer [140,141]. They have yet to be approved for clinical use, but they comprise a promising target for treating breast cancer and especially the refractory hormone-positive subtypes. HDACi prevent breast tumor progression via transcriptional inhibition of EMT-related genes, modulation of human epidermal growth factor receptor 2 (HER2) expression or induction of estrogen receptor (ER) in hormone receptor-negative tumors, as well as by increasing the sensitivity of hormonal therapy in ER positive tumors [142]. Only one phase III clinical trial exists that demonstrates the anti-tumor effects of HDACi in breast cancer [143]. HDACi have also been shown to target metastatic triple-negative breast cancer [144].

HMT inhibitors targeting PRMT3-6 in breast cancer have also been used preclinically [145], with PRMT5 inhibition displaying the greatest effects among the other family members. Its inhibition seems to reduce the metastatic potential and proliferation rate of breast cancer cells [146] and influence breast cancer sensitivity to other drugs [80]. On the other hand, PRMT2 methyltransferase itself was shown to improve the sensitivity of tamoxifen in ER positive breast cancer cells by transcriptionally suppressing the 36 kDa variant of estrogen receptor a, ER-a36 [147]. Similarly, PRMT4 activation was inhibited in endocrine resistant breast cancer cells [148].

Furthermore, EZH2 methyltransferase inhibitors have been used in clinical trials [149] and can be combined with Extracellular Signal-Regulated Kinase (Erk) inhibitors to suppress TGF-β-induced EMT [150]. The EZH2 inhibitor Tazemetostat, is currently under clinical trial in lymphomas and other tumors [151]. However, a recent clinical study of the EZH2 inhibitor GSK2816126 on lymphomas, solid tumors and multiple myelomas showed little effect in treatment efficiency and was terminated [152].

The non-selective Suppressor Of Variegation 3-9 Homolog 1 (SUV39H1) methyltransferase inhibitor, chaetocin [153] has been demonstrated to restore E-cadherin expression and the tumor suppressor p15INK4B, by reducing the levels of H3K9me3 on the promoters of these genes [154]. BIX01294, another histone methyltransferase inhibitor was shown to induce E-cadherin expression through inhibition of G9a and G9a-Like Protein (GLP) and reduction of H3K9me2 on its promoter [155,156]. UNC0638, another specific inhibitor of G9a and GLP methyltransferases with lower toxicity profile compared to BIX01294, was shown to exert similar effects and reduce the global H3K9me2 levels in in vivo [157].

The demethylase LSD1 implicated in the promotion of EMT can be also targeted to suppress metastasis. In vivo and in vitro studies showed that LSD1 inhibition by parnate or by an inhibitor targeting LSD1-SNAI1 interaction, leads to decreased cancer motility and invasiveness while also increasing E-cadherin [158]. Similar results were observed with the LSD1 inhibitor pargyline [159,160]. On the contrary, another clinical study on the LSD1 inhibitor GSK2879552 in patients with relapsed/refractory small cell lung carcinoma showed that the risk benefit was not significant to justify further studies on this drug [161]. However, more LSD1 inhibitors are currently tested in clinical trials for evaluation of their efficacy [162].

### 6.3. DNA Methylation Inhibitors

DNA methylation has also been explored as a possible therapeutic target for metastatic cancers. The DNA methylation status of cancer cells can predict their responsiveness to treatment with cisplatin, a standard chemotherapeutic agent used in gastric cancer. Furthermore, the DNA methylation inhibitor, decitabine applied in myelodysplastic syndromes (MDS) and acute myeloid leukemia (AML) has been shown to induce changes in cancer cell morphology, colony formation, and cell differentiation markers’ expression [163]. The hypomethylating agent 5-aza-2′-deoxycytidine can influence the differentiation status of cultured cancer cells and inhibit the High Mobility Group AT-Hook Protein 2 (HMGA2)-induced EMT [164,165,166]. 5-aza-2′-deoxycytidine was also able to restore epithelial phenotypes and inhibit EMT by promoting E-cadherin re-expression [167]. Moreover, 5-aza-2′-deoxycytidine-mediated targeting of BMP-4 that is epigenetically upregulated in cisplatin-resistant gastric cancer cell lines, was shown to improve their sensitivity to chemotherapy [168]. However, this effect of DNA demethylating agents, although capable of restoring genes silenced in cancer is not specific, and may result in the aberrant expression of many non-epithelial genes, affecting positively EMT progression and tumor metastasis [169].

### 6.4. NcRNAs

Lastly, the use of non-coding RNAs has been proposed as a treatment modality to reduce cancer metastasis. miRNAs and lncRNAs can decrease the expression of EMT-promoting transcription factors as well as the activity of epigenetic modifying enzymes. Therefore, increasing the expression of these non-coding RNAs or reintroducing them in the organism are under study. Despite the lack of in-depth knowledge, preclinical studies demonstrate promising results for future implementation of these epigenetic modulators in the treatment of cancer [170].

**Table 1:** Drug targeting of histone modifications.

Drug Category		Drug Name	Cancer Type	Effect	Reference
LMW EMT inhibitors	BRD4 Inhibitors	MS417	Colorectal	Inhibits metastasis via induction of E-cadherin and inhibition of Vimentin	[121]
		JQ1	Breast	Blocks BRD4 binding to K73/K76Ac2 on the TWIST transcription factor, resulting in decreased WNT5A expression and inhibition of EMT and metastasis	[122]
	Histone binder inhibitor	UNC3866	Prostate	Blocks the binding of CBX4/7 to methyl-lysine; unclarified role in EMT reversal	[124]
	CSC targeting drugs	Salinomycin	Breast, GI, Leukemia, Lung, Prostate	Influences the Twist-Snail/ZEB-E-cadherin axis and Wnt-TGF-β-BMP to inhibit EMT	[126,127,128,129,130]
Histone-modifying drugs	DNMT Inhibitors	Chaetocin	AML	Restores E-cadherin and p15INK4B expression by reducing SUV39H1- mediated H3K9me3 on their promoters	[154]
		BIX01294	HeLa cells	Induces E-cadherin expression by inhibiting G9a and GLP-mediated H3K9me2 deposition on its promoter.	[155,156]
		UNC0638	Pancreatic	Induces E-cadherin expression by inhibiting G9a and GLP-mediated H3K9me2 deposition on its promoter.	[157]
		Zebularine	Pancreatic	Used in combination with SAHA to promote the differentiation of cancer cells	[137]
	HDAC Inhibitors	SAHA	Pancreatic	Combined with Zebularine in order to promote differentiation of cancer cells	[137]
		Panobinostat	Hepatocellular	Inhibits cancer proliferation and induces apoptosis of cancer cells. Also increases differentiation markers in vivo	[135]
		Mocetinostat	Pancreatic	Specific antagonism of ZEB1-mediated silencing of miR-203, which is associated with tumor recurrence	[139]
		Entinostat	Breast	In phase III clinical trial	[143]
	PRMT5 Inhibitors	shRNA- mediated inhibition	Breast, Lung	Reduces metastatic potential, EMT and proliferation rate of cancer cells	[80,146]
	EZH2 Inhibitors	Tazemetostat	Advanced solid tumors and lymphomas	Under clinical trial investigation	[152]
		GSK2816126	Lymphomas, solid tumors and MM	Clinical study terminated due to little effect in treatment efficiency	[152]
	Erk Inhibitors	AZD6244	Breast	Combination with EZH2 inhibitors suppresses TGF-β-induced EMT	[150]
	LSD1 Inhibitors	Parnate	Myelogenous leukemia	Decreases motility and invasiveness, increases E-cadherin	[158]
		Pargyline	Lung	Suppresses proliferation, migration and invasion of cancer cells	[159,160]
		GSK2879552	Lung	Clinical study discontinued	[161]
DNA methylation inhibitors	Decitabine		MDS, AML	Changes cancer cell morphology, differentiation markers and inhibits proliferation	[163]
	5-aza-2′-deoxycytidine		Gastric	Improves sensitivity to chemotherapy, restores epithelial phenotypes by promoting E-cadherin re-expression and inhibits EMT	[167,168]

LMW; Low molecular weight, EMT; Epithelial to Mesenchymal Transition, BRD4; Bromodomain-containing protein 4, CSC; Cancer stem cell, GI; Gastrointestinal, WNT5A; Wnt Family Member 5A, CBX4/7; Chromobox 4/7, DNMT; DNA Methyltransferase, HDAC; Histone Deacetylase, SUV39H1; Suppressor Of Variegation 3-9 Homolog 1, G9a; Euchromatic histone-lysine *N*-methyltransferase 2 (EHMT2), GLP; G9a-like protein, ZEB1; Zinc finger E-box-binding homeobox 1, PRMT5; Protein Arginine Methyltransferase 5, EZH2; Enhancer of zeste homolog 2, MM; Multiple Myeloma, MDS; Myelodysplastic Syndromes, AML; Acute Myeloid Leukemia.

## 7. Conclusions—Future Perspectives

Collectively, metastasis is a critical step in cancer progression and worsens drastically patients’ survival. Its complexity stems from the wide variety of crosstalk pathways, as well as the heterogeneity observed in different cancer types and subtypes that either promote or inhibit the process. The subdivision of metastasis in three major steps has helped research to focus more on the specific checkpoints that regulate the process and elucidate previously unknown regulatory mechanisms for potential targeting.

Epigenetic modifications have recently emerged as critical regulators and drivers of the metastatic process. Targeting of these pathways holds a great therapeutic potential, with scientists being able not only to determine the epigenetic alterations, but also use newly discovered epigenetic modulators, to specifically target and reverse them. In this way, an effort is made to reset the process of metastasis and reconstitute the loss of epithelial-like phenotype in cancer cells. Until recently, most drugs targeting the epigenetic state of cells were only evaluated in vitro or in in vivo models. However, the constant progress in the field of epigenetics, has allowed their future implementation in clinical trials and in the clinical setting as individualized therapies that specifically target each cancer’s unique “epigenome”.

Despite these encouraging results, epigenetic therapies need extensive investigation to overcome the current lack of specificity of some agents, to elucidate the exact role of the different epigenetic alterations, and to determine the therapeutic effect of epigenetic regulation and the way to implement them in treatment schemes as a monotherapy or in conjunction with other agents. Future research should focus on unraveling the complex regulatory network induced by epigenetic mechanisms along with tumor microenvironment, aiming to define the best therapeutic targets and biomarkers for evaluating tumor aggressiveness and potential response to therapy. In these efforts, new technologies such as ATAC-seq, single-cell RNA-seq, and ChIP-seq will also prove invaluable and help scientists shed light in the entirety of the epigenome, its alterations in cancer, and the ways it can be targeted to increase patient prognosis, survival, and quality of life.

## Data Availability

Not applicable.

## Abbreviations

EMT	Epithelial to Mesenchymal Transition
P300/CBP	E1A Binding Protein P300/CREB Binding Protein
ZEB1	Zinc Finger E-Box Binding Homeobox 1
BRG1	BRM/SWI2-Related Gene 1
CtBP	C-terminal-binding protein
HDAC	Histone deacetylase
SNAI1	Snail Family of zinc finger proteins 1
SNAI2	Slug
JMJ3	Jumonji Domain Containing 3 Histone Lysine Demethylase
G9a	Euchromatic Histone Lysine Methyltransferase 2
SIN3A	SIN3 Transcription Regulator Family Member A
SET8	Lysine Methyltransferase 5A
H	Histone
K	Lysine
me	methylation
ac	acetylation
R	Arginine
TWIST1	Twist Family BHLH Transcription Factor 1
EZH2	Enhancer of Zeste 2 Polycomb Repressive Complex 2 Subunit
PRC2	Polycomb Repressive Complex 2
FZD7	Frizzled Class Receptor 7
FLIP	CASP8 and FADD Like Apoptosis Regulator
JMJD2B	Jumonji Domain-Containing Protein 2B
PHF8	PHD Finger Protein 8
NDRG1	N-Myc Downstream Regulated 1
GLI1	Glioma-Associated Oncogene Homolog 1
SMO	Smoothened, Frizzled Class Receptor
FOXF1	Forkhead Box F1
Bmi1	Polycomb Complex Protein BMI-1
SIRT2	Sirtuin 2
TGFBR2	TGF-β Receptor type 2
GCN5	General Control of Amino Acid Synthesis Protein 5-Like 2
PCAF	P300/CBP-Associated Factor
TIP60	Tat Interacting Protein, 60 kDa
DDR	DNA Damage Response
hMOF	Males-absent-on-the-first histone acetyltransferase
TMS1	Target of Methylation-induced Silencing
ESR1	Estrogen Receptor 1
Smad2	Mothers Against Decapentaplegic Homolog 2
EPCAM	Epithelial Cell Adhesion Molecule
ST14	Suppression of Tumorigenicity 14
ESRP1	Epithelial Splicing Regulatory Protein 1
RAB25	RAB25, Member RAS Oncogene Family
FLIP	CASP8 And FADD Like Apoptosis Regulator
PRMT	Protein Arginine Methyltransferase
MEP50	Methylosome Protein 50
GAS1	Growth Arrest Specific 1
PRC	Polycomb Repressive Complex
KLF2	Kruppel Like Factor 2
JARID	Jumonji, AT Rich Interactive Domain
TRIM33	Tripartite Motif Containing 33
Smad2/3	Mothers Against Decapentaplegic Homolog 2/3
HP1	Heterochromatin Protein 1-Alpha
GSC	Goosecoid Homeobox
MIXL1	Mix Paired-Like Homeobox
WDR5	WD Repeat-Containing Protein 5
KDM6B	Lysine (K)-Specific Demethylase 6B
ITG	Integrin
UTX	Ubiquitously- Transcribed X Chromosome Tetratricopeptide Repeat Protein
MMP-9/11	Metalloproteinase-9/11
SIX1	Sine Oculis Homeobox Homolog 1
MLL4	Mixed-Lineage Leukemia Protein 4
ROCK kinase	Rho-Associated Protein Kinase 1
FOXC1	Forkhead Box C1
SETDB1	SET Domain Bifurcated Histone Lysine Methyltransferase 1
CDH1	Cadherin 1
AXIN2	Axis Inhibition Protein 2
NKD1	Naked1
PPP2R2B	Protein Phosphatase 2 Regulatory Subunit 2 beta
PRICKLE1	Prickle Planar Cell Polarity Protein
SFRP5	Secreted Frizzled Related Protein 5
RKIP	Raf Kinase Inhibitory Protein
